# Targeting TOR dependence in cancer

**DOI:** 10.18632/oncotarget.110

**Published:** 2010-05-10

**Authors:** Matthew R. Janes, David A. Fruman

**Affiliations:** ^1^*Department of Molecular Biology & Biochemistry, and Institute for Immunology, University of California, Irvine, Irvine, CA, USA*

## Abstract

A challenge in cancer therapy has been to identify targets whose function is essential for survival of malignant cells but not normal cells. This Perspective discusses recent evidence that novel inhibitors of the kinase TOR can provide an unprecedented balance of anti-cancer efficacy and tolerability.

## INTRODUCTION

Dysregulation of cell growth and proliferation have been commonly linked to phosphoinositide 3-kinase (PI3K) and its downstream signaling effectors, which include the serine/threonine kinases AKT and target of rapamycin (TOR). Through activating mutations in PI3K or AKT, inactivating mutations in the PTEN tumor suppressor, or other mechanisms, there are many routes to augment PI3K/AKT/TOR signaling that promote cardinal hallmarks of malignant transformation [1]. A worldwide effort in academic and biopharma laboratories has resulted in a number of new therapeutic strategies to target one of more components of this complex signaling network [2-7]. Several small molecule inhibitors have shown impressive preclinical efficacy and are now in clinical trials. However, it has not been clear which of these approaches will best suppress oncogenic signaling while sparing normal cell homeostasis.

**Fig. 1:**
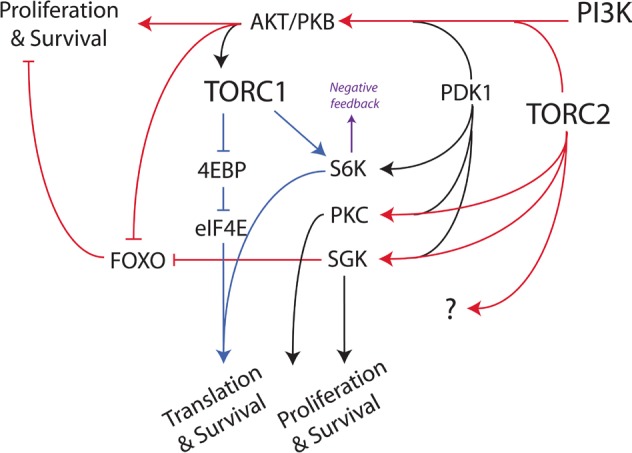
Simplified diagram of the PI3K/AKT/TOR signaling network. Red indicates TORC2-dependent steps. Blue indicates TORC1-dependent steps. The arrow between AKT and TORC1 represents a multistep process, in which activated AKT and other inputs from growth factor signaling pathways and nutrients are integrated to control TORC1 activity. Activated S6K mediates feedback inhibition of upstream signaling through several mechanisms.

TOR is a conserved Ser/Thr kinase that integrates both extracellular and intracellular signals to regulate cell growth, protein translation and metabolism [8-10]. Mammalian TOR (often termed mTOR) exists in two functionally distinct multi-protein complexes, TOR complex 1 (TORC1) and TOR complex 2 (TORC2). TOR kinase interacts with RAPTOR, LST8, FKBP38, DEPTOR and PRAS40 to form TORC1, or with RICTOR, LST8, SIN1, DEPTOR and PROTOR to form TORC2. The complexity of the signaling network is illustrated by the fact that TORC1 functions downstream of AKT, whereas TORC2 functions upstream (Fig. 1). Recent evidence indicates that both TORC1 and TORC2 function to orchestrate and maintain the excessive proliferative demands of tumorigenic cells [11-14].

Within the last year, a series of ATP-competitive catalytic site TOR inhibitors (TORC1/2 kinase inhibitors) have been developed, and compared to rapamycin (and “rapalogs”) that use an allosteric-based mechanism to inhibit TOR [15-21]. These reports strongly support the conclusion that TORC1/2 kinase inhibitors provide an improved strategy to target the PI3K/AKT/TOR network for therapeutic benefit in cancer.

### Mechanistic differences of TORC1/2 kinase inhibitors and rapalogs

TORC1 is an essential sensor for amino acids, oxygen, energy, and growth factor signaling [8-10]. When conditions are favorable for cell growth and division, TORC1 integrates these signals to promote mRNA translation, ribosome biogenesis and glycolytic metabolism. Two notable TORC1 substrates are S6K1 (on Thr389) and 4EBP1 (on several sites) (Fig. 1). Phosphorylation of S6K1 activates the enzyme, leading to increased phosphorylation of the S6 ribosomal protein and other substrates that regulate translation. Phosphorylation of 4EBP1 blocks its function as a suppressor of the initiation factor eIF4E. Rapamycin disrupts the TORC1 complex and partially inhibits TORC1 activity, with greater effects on phosphorylation of S6K than 4EBP1 [22-24]. This is an important distinction because of emerging evidence that 4EBP1 inhibition is a crucial gatekeeper of regulated mRNA translation and is more important than S6K for cellular transformation [12, 14]. TORC2 is activated through unknown mechanisms, and is insensitive to nutrients, energy or acute rapamycin treatment. TORC2 regulates a subgroup of AGC family kinases (Fig. 1), which include AKT, SGK (serum– and glucocorticoid–induced protein kinase), and PKC (protein kinase C), by phosphorylating the hydrophobic and turn motifs [25-28]. Genetic ablation of TORC2 (via deletion of rictor or Sin1) has significant impact on metabolic tissues [29-31] but seems to be selectively toxic to cancer cells compared to normal cells [11, 16, 17, 19, 26].

Rapamycin and rapalogs (everolimus, temsirolimus) can slow the proliferation of cancer cell lines and have achieved some success in specific malignancies [23, 32]. Unfortunately, however, their overall efficacy as cancer therapeutics has been limited. The major drawbacks of rapalogs are: 1) S6K is exquisitely inhibited, yet the control of 4EBP and mRNA translation is far less sensitive [23, 24]; 2) TORC2 activity is not acutely blocked (though it can be suppressed upon sustained exposure [33]); 3) the loss of a feedback inhibition pathway mediated by S6K results in amplified PI3K signaling, with potential to amplify RAS, MAPK, and TORC2 itself [34-38]. In addition to these drawbacks, cell-extrinsic factors have been reported to prompt rapalog resistance in the clinical setting of recurrent PTEN-deficient glioblastomas [39]. To overcome these drawbacks, the pursuit of selective TOR kinase inhibitors has been a strong priority [23, 40]. ATP-competitive TOR kinase inhibitors that also inhibit PI3K and other enzymes have been studied for decades, exemplified by the highly nonselective compound LY294002 and the more refined panPI3K/TOR inhibitors PI-103 and BEZ-235 [3, 6, 7]. These compounds generally have stronger anti-cancer activity than rapalogs, but strong PI3K inhibition might be a liability when considering toxicity to normal cells (see below).

A report from the Shokat group was the first to describe ATP-competitive inhibitors that selectively inhibit TOR, and to document the mechanistic differences between TOR kinase inhibitors and rapamycin [16]. Soon after, several other groups confirmed these findings using TOR kinase inhibitors with distinct chemical scaffolds [15, 17, 19-21]. In each case, the active-site inhibitor completely blocked TORC1 signaling (both S6K^Thr389^ and 4EBP1^Thr37/46^ ^and Ser65^) and TORC2 signaling (AKT^Ser473^). In fibroblasts, muscle cells and solid tumor cell lines, the inhibition of rapamycin-resistant TOR outputs was associated with stronger suppression of protein synthesis and cell proliferation, and greater impact on cell size and metabolism. Possible mechanisms for differential effects on mRNA translation and metabolism were reviewed recently [40]. Supporting the selectivity of TORC1/2 kinase inhibitors, none of the compounds strongly reduced phosphorylation of AKT on Thr308, the activation loop site that is phosphorylated in a PI3K-dependent manner by phosphoinositide-dependent kinase-1 (PDK-1).

In fibroblasts, the anti-proliferative effects of TORC1/2 kinase inhibitors were entirely attributable to TORC1 inhibition. Thus, cells lacking TORC2 components proliferated normally yet retained full sensitivity to TORC1/2 kinase inhibitors [16, 17, 19]. The relative importance of TORC1 vs. TORC2 inhibition for suppression of cancer cell proliferation and survival is not yet clear, and might be dependent on cell context. If TORC2 inhibition contributes to the mechanism, it will be important to determine which TORC2 substrates are the relevant mediators of cancer cell growth and survival. Although much attention has focused on TORC2-dependent phosphorylation of AKT (on Ser473), regulation of SGK and PKC might be of crucial importance in some contexts. With PDK-1-dependent phosphorylation of AKT^T308^ intact, AKT remains partially active in TORC2-deficient cells [41, 42]. In contrast, TORC2-dependent phosphorylation of the SGK hydrophobic loop is a prerequisite for SGK phosphorylation on the activation loop [26]. The SGK family (SGK1, SGK2, SGK3) of AGC kinases share strong (>50%) homology with the AKT kinase domain. SGK1 shares substrate preference for FOXO transcription factors (Fig. 1), a well-described target of AKT [43]. SGK3 plays a role in AKT-independent survival in a variety of cancers driven by activating mutations in PI3K [44]. Therefore, SGK may be a key target of TORC2 and the potency attributed to TORC1/2 kinase inhibitors could be due in part to diminished SGK activation. PKC activity might also contribute to tumor growth [45].

An interesting twist to TOR biology was the discovery of DEPTOR as an endogenous inhibitor of both mTOR complexes [46]. Peterson, Sabatini and colleagues tested whether overexpression of DEPTOR could phenocopy cells treated with TORC1/2 kinase inhibitors. They observed that a prominent effect of DEPTOR was to augment AKT activity through disabling of the negative feedback loop mediated by S6K. The rebound activation of PI3K was strong enough to override DEPTOR's inhibitory effect on TORC2, such that AKT phosphorylation on Ser473 was maintained. They further showed that prolonged treatment of cells with suboptimal doses of TORC1/2 kinase inhibitors also elevated AKT phosphorylation. These findings emphasize that when TORC1 activity is reduced, any remaining TORC2 activity can phosphorylate AKT and other substrates that are co-regulated via PI3K/PDK-1. In other words, a complete and optimal amount of TORC1/2 inhibition might need to be achieved and maintained to prevent rebound activation of PI3K signaling. Consequently, it will be important to monitor the degree of PI3K function and downstream signaling in cells and tissues exposed to different doses of TORC1/2 inhibitors.

### Efficacy of TORC1/2 kinase inhibitors *in vivo*

Several TORC1/2 kinase inhibitors have been tested in cancer models *in vivo*. The first publication, from a group at Wyeth, showed that the compound WYE-354 could delay growth of U87MG tumors in nude mice [21]. Lead optimization by this group led to the discovery of the compound WYE-125132 (abbrev. WYE-132), which showed strong single-agent activity in a range of xenograft models representing various solid tumor types [20]. The anti-tumor effect of WYE-132 was markedly stronger than that achieved by the rapalog CCI-779. In some models, regression was observed. Similarly, the TORC1/2 kinase inhibitor AZD8055 (developed by AstraZeneca) demonstrated growth inhibition and/or regression in xenograft models [15]. In each of these studies, *in vitro* experiments confirmed that TORC1/2 kinase inhibitors block rapalog-resistant outputs of TORC1 and TORC2 across a range of cancer cell lines. Interestingly, WYE-354 failed to inhibit protein synthesis in the colon cancer cell lines HCT116 and HT29 and this was associated with absence of pro-apoptotic effects [21, 40]. Further study of cell lines and primary specimens that are resistant to TORC1/2 inhibitors might provide biomarkers that can be used to predict efficacy.

Our group compared the TORC1/2 kinase inhibitor PP242 to rapamycin in acute leukemia models driven by the BCR-ABL fusion tyrosine kinase encoded by the t(9;22) Philadelphia (Ph) chromosomal translocation [18]. As in studies of fibroblasts and solid tumor cell lines, we found that PP242 blocked rapamycin-resistant TORC1 and TORC2 signaling outputs in mouse and human leukemia cells representing either Ph^+^ B-precursor acute lymphoblastic leukemia (B-ALL) or chronic myeloid leukemia (CML). PP242 did not alter cellular levels of PIP3, a measure of PI3K activity. *In vitro*, PP242 caused cell cycle arrest similar to rapamycin, but also triggered apoptosis. In a mouse syngeneic model of Ph^+^ B-ALL, PP242 prolonged survival whereas rapamycin had no protective effect. In these experiments we also compared PP242 to compounds representing the panPI3K/TOR target profile. Notably, we found that the efficacy of PP242 *in vitro* and *in vivo* was comparable to PI-103 or BEZ-235. These results indicate that PI3K inhibition is dispensable for strong anti-leukemic efficacy when both TOR complexes are fully suppressed. PP242 also slowed growth and caused apoptosis *in vivo* when tested in a mouse thymoma model [12]; rapamycin had no protective effect in this model.

In patients with chronic phase CML, impressive therapeutic responses have been achieved with BCR-ABL tyrosine kinase inhibitors (TKIs) [47]. Unfortunately, TKI resistance often develops and these agents fail to achieve durable remission in later phases of CML (i.e. blast crisis) or in patients with Ph^+^ B-ALL [48]. We compared the ability of various TOR inhibitors to augment the efficacy of BCR-ABL TKIs. Although functional synergy was achieved with all combination approaches, PP242 was more effective than rapamycin when combined with imatinib or dasatinib, both *in vitro* and *in vivo*. In mice bearing xenografts of the Ph^+^ B-ALL cell line SUP-B15, dasatinib plus PP242 caused leukemia regression whereas dasatinib plus rapamycin only slowed expansion. In xenografts of primary human Ph^+^ B-ALL specimens, dasatinib plus PP242 caused significantly greater inhibition of leukemia cell proliferation compared to dasatinib alone. At the signaling level, PP242 suppressed TOR outputs more thoroughly than rapamycin even in the presence of BCR-ABL TKIs, probably because cell-extrinsic factors provide an alternative oncogene-independent route to TOR activation. These results provide proof-of-concept that encourages further testing of TORC1/2 kinase inhibitors in combination with TKIs in other tumor settings (to avoid confusion, we oppose a recent suggestion that TORC1/2 kinase inhibitors be abbreviated "TKIs" [40]). TORC1/2 kinase inhibitors also have broader anti-angiogenic impact than rapalogs [20] and could enhance the efficacy of existing angiogenesis inhibitors. Indeed, in a renal cell carcinoma model the compound WYE-132 showed greater ability than CCI-779 to synergize with bevacizumab (Avastin), a monoclonal antibody to VEGF-A [20]. These findings highlight the clinical potential of TORC1/2 kinase inhibitors for combinatorial therapies. Careful strategies will need to be employed when developing a dosing regimen to best obtain the full therapeutic benefit of drug combinations.

### Tolerability of TORC1/2 kinase inhibitors in vivo

TOR was first discovered as the molecular target of the immunosuppressive drug rapamycin. In fact, much of what we know about mTOR and its roles in immunological function has derived from experiments using rapamycin. It is now clear that rapamycin suppresses T and B cell proliferation, and promotes tolerance induction through at least three mechanisms: induction of T cell anergy, generation of regulatory T cells (Tregs), and impairment of dendritic cell maturation [49-51]. Paradoxically, rapamycin enhances the generation and quality of CD8 T cell memory [52, 53], and also potentiates inflammatory responses of innate immune cells [49-51]. In addition to these immune effects, systemic rapamycin treatment in humans results in other significant toxicities [54].

**Fig. 2:**
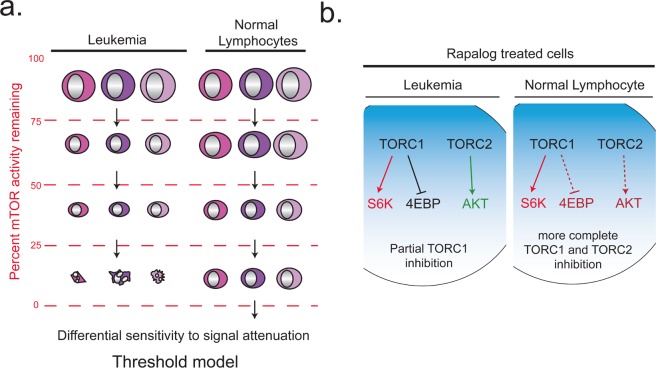
Working models to explain selective effects of TORC1/2 kinase inhibitors on leukemia cells (A) and rapalogs on normal lymphocytes (B) The threshold model **(A)** posits that leukemia cells depend on a higher output of mTOR signaling for growth and survival. Leukemia cells commit to cell death process when TORC1 and TORC2 are strongly (or transiently) suppressed, whereas normal lymphocytes grow more slowly but do not die. At intermediate levels of mTOR signaling, leukemia cells exhibit slower growth whereas normal cells are unaffected. The scheme in panel **(B)** proposes that in normal lymphocytes, rapamycin and analogs inhibit TORC1 and TORC2 more effectively than in leukemia cells. Because rapalogs act through an allosteric mechanism, the magnitude and kinetics of their effects might differ depending on the composition and turnover of TOR complexes in distinct cell contexts. We reported experimental evidence to support the threshold model in (A) [18], but further work is necessary to test the model in (B).

Given the more complete suppression of TOR signaling by ATP-competitive inhibitors, one might expect these compounds to have even more severe toxicities and immunomodulatory activities. However, the evidence so far indicates that this is not the case. Several TORC1/2 kinase inhibitors have entered clinical trials, indicating that animals tolerate these compounds at doses that produce therapeutic effects. In our leukemia models, we noted that PP242 was not toxic to normal mouse bone marrow cells under conditions where human Ph^+^ B-ALL cells disappear [18]. In fact, proliferation of endogenous bone marrow cells returned in mice treated with dasatinib plus PP242. *In vitro*, PP242 caused hematotoxicity only at concentrations much higher than those needed to cause leukemia cell death. Remarkably, PP242 and the structurally unrelated TORC1/2 inhibitor Ku-0063794 had little impact on the proliferation of activated lymphocytes, whereas these responses were strongly suppressed by rapamycin. Mice treated with rapamycin showed a disruption of lymphoid architecture and a near-complete block in T cell-dependent antibody responses. These effects were not observed in mice treated with PP242 at doses showing profound suppression of leukemia. Importantly, the panPI3K/TOR inhibitor PI-103 showed less selectivity for leukemia cells and was reported by another group to be immunosuppressive [55]. Thus, selective TORC1/2 kinase inhibition provides a favorable tolerability profile compared to rapamycin or panPI3K/TOR inhibitors.

It is interesting to speculate on the mechanism of selective anti-cancer effects of TORC1/2 kinase inhibitors. Mouse models of PTEN-deficient prostate cancer support the idea that cancer cells with elevated PI3K signaling are uniquely addicted to TORC2 [11, 13]. One study showed that simultaneously deleting *Pten* and *mTor* in the prostate epithelium suppressed prostate cancer development while sparing the morphology and function of normal prostate tissue [13]. The suppression of neoplasia was more pronounced than with 4–week pharmacological treatment with the rapalog RAD001. This observation does not distinguish whether the PTEN-deficient cells relied on rapalog-resistant outputs of TORC1 or TORC2. However, another group obtained similar results by deleting *Rictor* in the prostate epithelium, showing that *Pten*-null driven prostate cancer progression requires TORC2 function [11]. In support of this conclusion, knockdown of rictor expression suppressed the development of a *PTEN*-null PC-3 human prostate cancer xenograft model. The preservation of normal tissue in the absence of TORC2 is consistent with studies of mouse embryo fibroblasts (MEFs). In MEFs, loss of rictor or Sin1 does not affect cell proliferation and the cells remain equally sensitive as wild-type MEFs to growth suppression by TORC1/2 kinase inhibitors [16, 19]. On the other hand, the fact that complete TORC1 inhibition strongly suppresses growth factor-dependent proliferation of MEFs implies that TORC1/2 kinase inhibitors should have negative impacts on mitotic tissues. Indeed, we found that PP242 did suppress hematopoietic colony formation and lymphocyte proliferation at a high concentration (1μM) [18]. We propose a “threshold” model in which normal cells can tolerate a lower output of TORC1 and TORC2 signaling than transformed cells (Fig. 2A). In some cell types, such as prostate epithelium, *mTor* expression appears completely dispensable [13]. In other cell types, exemplified by T lymphocytes, deletion of *mTor* delays but does not fully arrest proliferation [56]. In support of the threshold model, mice heterozygous for a kinase-dead allele of *mTor* show no impairment of T cell proliferation [57].

Perhaps a greater puzzle is why rapamycin seems more potent at suppressing normal lymphocytes than leukemia cells or solid tumor cell lines. It is possible that lymphocyte signaling complexes are wired differently, such that rapamycin suppresses TOR outputs that are resistant to the drug in other settings (Fig. 2B). There is evidence that the magnitude and kinetics of rapamycin's effect on 4EBP1 phosphorylation and TORC2 function are cell type-dependent [22, 33]. Alternatively, feedback effects of TORC1 inhibition might have severe impact in primary lymphocytes. There is also evidence that rapamycin disrupts noncatalytic scaffolding functions of TOR [58-60], whereas TORC1/2 kinase inhibitors do not affect the complexes [20]. It is worth testing these models in lymphocytes and in cancer cell lines that are particularly sensitive to rapamycin.

## FUTURE DIRECTIONS

TORC1/2 kinase inhibitors represent a major breakthrough in targeting the PI3K/AKT/TOR signaling network for cancer therapeutics. In order to realize the full clinical potential of these compounds, more basic research is required. Here we emphasize three objectives of primary importance.

First, we need a greater understanding of the mechanism of action of TORC1/2 kinase inhibitors in cancer cells. Which cellular processes regulated by TORC1 and/ or TORC2 are relevant to the therapeutic effects? These efforts will help identify biomarkers of drug efficacy and resistance. A recent study from the Ruggero group provided important insights in a mouse thymoma model driven by activated AKT [12]. This study showed that TORC1-dependent eIF4E hyperactivation (via 4EBP1 inactivation) was essential for tumor growth, whereas S6K activation was dispensable. In this model, the pro-apoptotic effect of PP242 was entirely dependent on the 4EBP1/eIF4E axis. However, it remains possible that TORC2 inhibition contributes to death of cancer cells that do not express a constitutively active AKT molecule. An interesting finding of Hsieh et al. was that regulated translation of the anti-apoptotic Bcl-2 family member MCL-1 might play a unique pro-survival role. MCL-1 was also found to be modulated by PI3K/TOR signaling in the setting of mutant epidermal growth factor receptor (EGFR) driven non-small cell lung cancers [61]. It will be interesting to determine whether TORC1/2 kinase inhibitors trigger cell cycle arrest also by controlling expression of specific proteins, or through general inhibition of translation.

Second, it will be important to determine the efficacy of TORC1/2 kinase inhibitors across a broad range of tumor types and driving mutations. Human cancers are heterogeneous, and xenograft models have already shown variable responses of solid tumors and primary leukemias [15, 18, 20]. Preclinical screens of drug efficacy can provide valuable information about which populations are most likely to benefit from any targeted agent. Identifying tumor characteristics that correlate with drug resistance will also be valuable, as understanding the molecular basis of resistance can lead to combination approaches that achieve greater efficacy. There might also be heterogeneity *within* tumors such that subpopulations, for example quiescent cells with stem-like properties, survive independently of TOR activity. A related issue is the need to test TORC1/2 kinase inhibitors in combination with current front-line therapies. It is likely that novel targeted agents will be tested first in clinical settings where standard therapy has failed, but could eventually be used as adjuvant therapy to augment the initial response. Our data provide strong justification for including TORC1/2 kinase inhibitors in clinical protocols that involve TKIs targeting BCR-ABL or other oncogenic kinases.

Third, we need to explore in greater detail how TORC1/2 kinase inhibitors affect immune function. Our published experiments with lymphocyte proliferation and antibody production have only scratched the surface of this important problem. TORC1/2 kinase inhibitors should be evaluated in various settings in which rapalogs have a profound impact: innate immune cell function, T and B cell differentiation, memory, and regulatory T cell induction. It is likely that drug concentration will have an important influence on functional outcomes, as illustrated by our studies of lymphocyte proliferation [18]. Similarly, the impact of TORC1/2 kinase inhibitors on immune function *in vivo* will likely depend on pharmacokinetics---and may therefore differ among different candidate molecules. An important complementary approach will be to study mice genetically deficient in individual mTOR complexes, to elucidate the separate roles of TORC1 versus TORC2 on innate and adaptive immune responses. One consequence of these studies might be that TORC1/2 kinase inhibitors have therapeutic value in certain immune-mediated diseases. In the setting of high-risk leukemias, it is essential to eradicate minimal residual disease (MRD) following chemotherapy regimens. One strategy to minimize MRD is allogeneic hematopoietic stem cell transplantation (allo-SCT), where the donor marrow attacks recipient MRD, also termed graft versus leukemia (GVL). It is interesting to note that many active clinical trials of rapalogs in cancer are testing their efficacy to suppress graft versus host disease (GVHD), a common allo-SCT complication. In the setting where rapamycin is used to suppress GVHD, will it also affect MRD by modulating GVL or through direct effects on the residual leukemia cells? Will TORC1/2 inhibitors achieve similar immunosuppression of GVHD, yet still achieve effective clearance of MRD? In solid tumor settings, will TORC1/2 kinase inhibitors have different effects than rapalogs on immunological tolerance? As TOR kinase-targeted therapies enter the clinical arena, it will be important to understand how they will best complement or interact with current clinical practices to harness immune responses and eradicate tumor cells.
